# Recent Advancements in Co_3_O_4_-Based Composites for Enhanced Electrocatalytic Water Splitting

**DOI:** 10.3390/mi15121450

**Published:** 2024-11-29

**Authors:** Komal S. Wagh, Sagar M. Mane, Aviraj M. Teli, Jae Cheol Shin, Jaewoong Lee

**Affiliations:** 1 Department of Fiber System Engineering, Yeungnam University, 280 Dehak-Ro, Gyeongsan 38541, Republic of Korea; 2 Division of Electronics and Electrical Engineering, Dongguk University-Seoul, Seoul 04620, Republic of Korea

**Keywords:** Co_3_O_4_, intrinsic features, material composites, bifunctional electrocatalysts

## Abstract

The pursuit of efficient and economical catalysts for water splitting, a critical step in hydrogen production, has gained momentum with the increasing demand for sustainable energy. Among the various electrocatalysts developed to date, cobalt oxide (Co_3_O_4_) has emerged as a promising candidate owing to its availability, stability, and catalytic activity. However, intrinsic limitations, including low catalytic activity and poor electrical conductivity, often hinder its effectiveness in electrocatalytic water splitting. To overcome these challenges, substantial efforts have focused on enhancing the electrocatalytic performance of Co_3_O_4_ by synthesizing composites with conductive materials, transition metals, carbon-based nanomaterials, and metal–organic frameworks. This review explores the recent advancements in Co_3_O_4_-based composites for the oxygen evolution reaction and the hydrogen evolution reaction, emphasizing strategies such as nanostructuring, doping, hybridization, and surface modification to improve catalytic performance. Additionally, it examines the mechanisms driving the enhanced activity and stability of these composites while also discussing the future potential of Co_3_O_4_-based electrocatalysts for large-scale water-splitting applications.

## 1. Introduction

With the depletion of non-renewable resources, such as fossil fuels, and escalating environmental challenges, exploring novel and innovative sustainable energy technologies has become increasingly urgent. In particular, recent decades have witnessed a growing emphasis on transitioning from carbon-heavy, non-renewable energy sources to clean energy alternatives. To achieve this shift, significant innovations are underway, focusing on the creation, storage, and conversion of clean energy [1,2,3]. Among these advancements, hydrogen energy technologies stand out as a promising solution to the global demand for cheap, efficient, dependable, and clean energy. The potential of these technologies to drastically reduce or eliminate energy-related emissions and environmental impacts further strengthens their appeal. Notably, hydrogen is 33% more energy-efficient than gasoline, and its combustion produces only water as a by-product. This clean energy carrier can be generated from various sources, including coal, water, hydrogen sulfide, solid waste, and hydrocarbons, through techniques such as gasification, electrolysis, hydrocarbon reforming, and partial oxidation [2,4,5].

Among these, water electrolysis or water splitting produces hydrogen by chemically separating the primary components of water, hydrogen, and oxygen. Given the abundance of water on Earth, this approach is particularly significant, as it offers a direct and sustainable pathway toward hydrogen production. Water electrolysis involves two primary reactions: the hydrogen evolution reaction (HER) and the oxygen evolution reaction (OER). These reactions are complex electrochemical processes that demand substantial energy to proceed efficiently. During the HER at the cathode, protons are reduced to form hydrogen gas, while the OER at the anode involves the oxidation of water to produce oxygen gas. While both these reactions are energy-intensive, the OER presents a more significant challenge owing to the energy required to break O–H bonds and release O_2_ [6,7].

Electrocatalysts are indispensable in water splitting, as they reduce the activation energy required for both the HER and OER, enabling these reactions at lower overpotentials and with greater efficiency. Without effective electrocatalysts, the energy demands for water splitting would be prohibitively high, rendering large-scale hydrogen production impractical. To address this challenge, researchers have investigated various materials as electrocatalysts for these reactions. Among these materials, platinum and ruthenium oxide (RuO_2_) have been recognized for their exceptional catalytic properties. However, the high costs and limited availability of these noble metals limit their use in large-scale commercial applications [8,9,10]. As an alternative, transition metal oxides, particularly cobalt oxide (Co_3_O_4_), have recently garnered considerable attention owing to their exceptional catalytic activity, distinct electrical properties, and natural abundance. Co_3_O_4,_ a spinel-type metal oxide with mixed valence states (Co^2+^ and Co^3+^), facilitates redox reactions essential for water-splitting processes. Furthermore, Co_3_O_4_ exhibits thermodynamic stability and functions effectively in both acidic and alkaline environments, making it a versatile electrocatalyst [11,12,13,14].

Despite these advantages, however, the intrinsic catalytic activity of Co_3_O_4_ remains inadequate for large-scale water splitting, particularly when compared to noble metal catalysts such as RuO_2_ or iridium oxide for the OER and platinum for the HER. Consequently, substantial efforts have been directed toward developing Co_3_O_4_-based composites to enhance catalytic performance for both the HER and OER [15,16,17,18,19,20,21]. Recent advancements in materials science have revealed that combining Co_3_O_4_ with conductive materials [22], carbon-based nanostructures [23,24,25,26], 2D materials [19,27,28,29], metal–organic frameworks (MOFs) [30], other metal oxides or sulfides [15,16,17,18,31], heteroatom doping [32,33], and composites fabricated by introducing very small quantity of various noble metals [34] can substantially improve its catalytic performance. These hybrid materials exhibit synergistic effects that enhance catalytic efficiency, improve electron transfer, and increase stability during water-splitting reactions.

For instance, recently, Li et al. [14] designed a substantially competent and durable electrocatalyst for acidic overall water splitting through the integration of platinum (Pt) into low-content ruthenium (Ru)-doped Co_3_O_4_. It is found that Pt incorporation enhances water-splitting performance by facilitating electron transfer, stabilizing Ru and Co in low valence states to prevent overoxidation and optimizing HER and OER energy barriers. Afterward, Wang et al. [32] used a simple method to improve the bifunctional (HER and OER) electrocatalytic activities of Co_3_O_4_. In this method, he adopted direct doping of heteroatom phosphorus (P), which effectively alters their electronic structure and enhances catalytic efficiency. Tian et al. [35] fabricated a CoP/Co_3_O_4_ heterojunction on titanium mesh (TM) using a straightforward in situ phosphating method. This heterojunction exhibited superior HER and OER performance compared to the individual single-phase materials which were ascribed to the lattice distortion realized with the addition of P atom in place of O atom.

Furthermore, to address the inherent limitations of pure Co_3_O_4_, such as low conductivity and restricted active site exposure, researchers have modified its surface properties and integrated complementary materials to create composite structures_._ Moreover, tuning the morphology and nanostructure of Co_3_O_4_-based composites has proven effective in exposing more active sites, thereby optimizing the adsorption and desorption of reactants and products, which are essential for boosting catalytic activity [11,13,36,37,38,39]. For example, Wang et al. [40] developed Co_3_O_4_/Co_3_S_4_@NF heterojunction catalysts featuring a nanoflower morphology. He utilized an ion etching technique to promote the oxygen and sulfur dual vacancies in this heterostructure. The vacancies enhance heterojunction conductivity, facilitating interfacial charge transfer and improving surface reactant adsorption and product desorption. Meanwhile, the oxygen and sulfur vacancies introduce local structural distortions, enrich active site availability, and modulate charge distribution for improved catalytic performance. Liu et al. [11] synthesized Co_3_O_4_ with varying facets and morphologies to investigate their influence on overall water-splitting efficiency. Through a combination of theoretical analysis and experimental evaluation, they established a correlation between the structural and morphological variations and the resulting electrocatalytic performance. Guo et al. [41] synthesized Co_3_O_4_ quantum dots using a straightforward solvothermal approach and investigated the influence of quantum confinement on the photocatalytic water-splitting process. The quantum confinement effect resulting from the small size of Co_3_O_4_ quantum dots was found to elevate their conduction band edge, enabling ligand-to-metal charge transfer from the 2p orbital of oxygen to the 3d orbital of Co^2+^. This charge transfer, essential for overall water splitting, is not feasible in bulk Co_3_O_4_ crystals.

Many previous studies on Co_3_O_4_ and its composites with various materials have primarily concentrated on either the hydrogen evolution reaction (HER) or the oxygen evolution reaction (OER), with significant progress in Co_3_O_4_-based composites for OER covered by Hu et al. [1] and for HER by Ma et al. [7]. Additionally, some reviews have addressed the broader advancements in Co-based catalysts for both HER and OER electrocatalysis [42,43]. However, there is a notable lack of comprehensive review specifically focused on Co_3_O_4_-based composites for bifunctional electrocatalytic activities. Hence through this review article, we aim to summarize the ongoing progress in Co_3_O_4_-based composites specifically simultaneously used for bifunctional electrocatalytic activities, including both hydrogen evolution reaction (HER) and oxygen evolution reaction (OER).

This review article provides a comprehensive overview of the recent developments in Co_3_O_4_-based composites for enhanced overall electrocatalytic water splitting. It explores the advantageous properties of Co_3_O_4_ that make it a promising metal oxide for electrocatalytic applications. Additionally, the review summarizes potential enhancements achieved in both the HER and OER through the use of nanostructured composites, surface modifications, and the incorporation of complementary materials. Finally, it highlights the challenges and future directions in developing Co_3_O_4_-based electrocatalysts, with an emphasis on designing cost-effective and efficient materials for large-scale hydrogen production. Overall, this review aims to offer insights into the potential of Co_3_O_4_-based composites as key components in future sustainable energy technologies.

## 2. Promising Features of Co_3_O_4_ Beneficial for Electrocatalytic Applications

The advantageous physical and chemical characteristics of Co_3_O_4_ make it promising for use in overall water-splitting applications. These properties make Co_3_O_4_ particularly effective as a catalyst for the HER and OER, which represent critical processes responsible for splitting water into hydrogen and oxygen. This section outlines the key features of Co_3_O_4_ that contribute to its potential as a material for electrocatalytic applications.

### 2.1. Mixed Valence States (Co^2+^/Co^3+^)

Co_3_O_4_ is notable for its ability to possess two distinct valence states: Co^2+^ and Co^3+^. These valence states appear in different coordination environments. Specifically, in the spinel crystal structure of Co_3_O_4_, Co^2+^ ions occupy tetrahedral sites, while Co^3+^ ions are positioned in octahedral sites. This unique arrangement of ions enables Co_3_O_4_ to efficiently mediate oxidation and reduction processes, which are essential for its catalytic efficacy in water splitting [12,44]. The coexistence of Co^2+^ and Co^3+^ ions facilitates efficient electron transfer, driving the oxidation and reduction reactions involved in water splitting [11,12,44,45,46]. Liu et al. [11] investigated the impact of different crystal facets on the overall electrocatalytic performance of Co_3_O_4_. Using a combination of theoretical and experimental methods, they evaluated the influence of various facets on HER and OER activities. The analysis revealed that facets with a higher density of dangling Co^3+^ bonds and greater surface energy exhibited the most favorable electrocatalytic properties. Specifically, the (111) crystal plane of Co_3_O_4_ was observed to possess the highest concentration of dangling Co^3+^ bonds and surface energy. Accordingly, among the four facets studied, the (111) facet demonstrated the highest electrocatalytic efficiency, with performance ranked in the order: (001) < (110) < (112) < (111). The overpotentials required for OER activity in an alkaline electrolyte (1 M KOH) at a current density of 10 mA cm^−2^ were measured as 362, 341, 312, and 285 mV for the (001), (110), (112), and (111) facets, respectively. A similar declining trend was observed for the HER activities, with overpotentials of 248, 217, 196, and 150 mV for the above facets.

**Figure 1:**
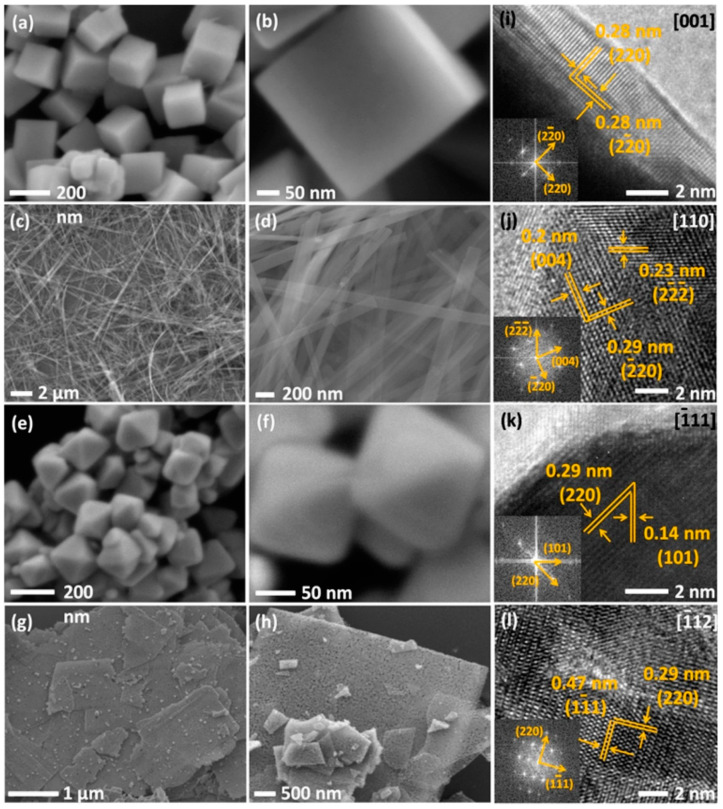
Morphological analysis of Co_3_O_4_ with different crystallographic facets: (**a**,**b**) Scanning electron microscopy (SEM) images depicting nanocubes with the [001] facet, (**c**,**d**) SEM images illustrating nanobelts with the [110] facet, (**e**,**f**) SEM images depicting nanooctahedra with the [111] facet, (**g**,**h**) SEM images presenting nanosheets with the [112] facet, and (**i**–**l**) high-resolution transmission electron microscopy images of the nanocubes, nanobelts, nanooctahedra, and nanosheets, respectively, confirming their crystallographic facets. Reproduced with permission from Liu et al. [11], Probing the crystal plane effect of Co_3_O_4_ for enhanced electrocatalytic performance toward Efficient overall water splitting; Applied Materials & Interfaces, Published by American Chemical Society, 2017.

**Figure 2:**
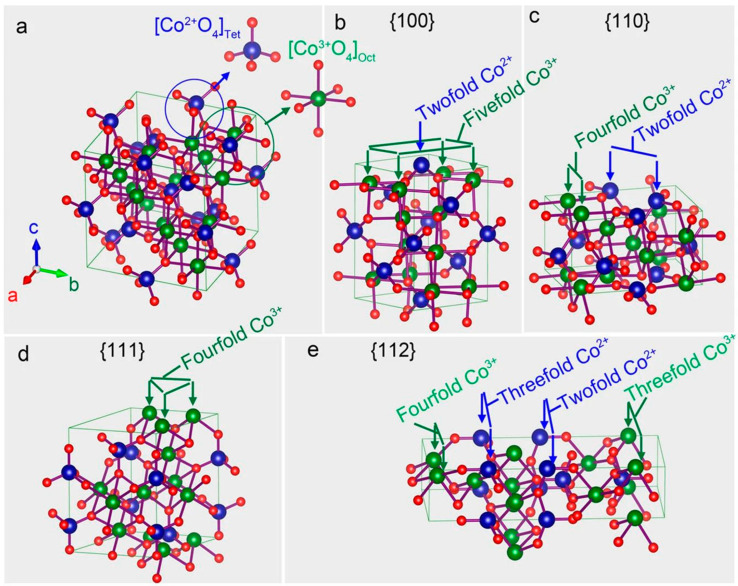
Crystallographic illustrations: (**a**) crystal structure of spinel Co_3_O_4,_ and (**b**–**e**) modifications within the crystal structure corresponding to individual facets of Co_3_O_4_: [001], [110], [111], and [112], respectively. Reproduced with permission from Su et al. [47], Single crystalline Co_3_O_4_ nanocrystals exposed with different crystal planes for Li-O_2_ batteries; Scientific Reports, Published by Springer Nature, 2014.

Figure 1a–h illustrates scanning electron microscopy (SEM) images of Co_3_O_4_ with different facets, highlighting their surface microstructures, while Figure 1i–l presents high-resolution transmission electron microscopy images, presenting crystallographic details about the facets using fast Fourier transform patterns. Notably, Co_3_O_4_ with the (111) facet exhibits a high specific energy capacity when applied in Li–O_2_ batteries [47]. This superior performance is attributed to the increased number of active sites for catalytic activity, facilitated by the higher density of dangling Co^3+^ bonds and the enhanced adsorption of ionized oxygen species owing to the facet’s high surface energy. These findings are further supported by the crystallographic illustrations of each facet, as presented by Su et al. [47] (Figure 2a–e).

**Figure 3:**
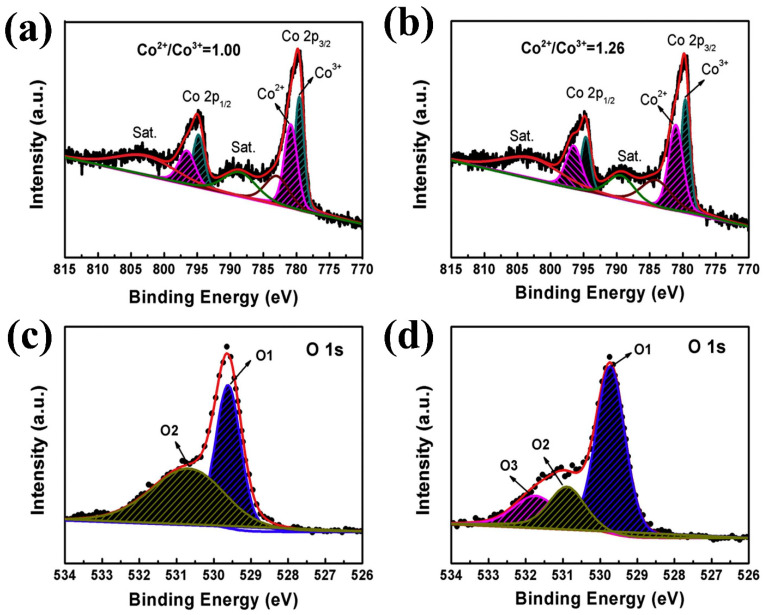
Results derived from the X-ray photoelectron spectroscopy analysis of Co_3_O_4_, demonstrating the impact of annealing atmosphere on the Co^2+^/Co^3+^ ratio: (**a**) Co2p spectra of the sample annealed in an air atmosphere, (**b**) Co2p spectra of the sample annealed in an O_2_-Ar atmosphere, (**c**) O1s spectra of the sample annealed in an air atmosphere, and (**d**) O1s spectra of the sample annealed in an O_2_-Ar atmosphere. Reproduced with permission from Zhu et al. [12], Special atmosphere annealed Co_3_O_4_ porous nanoclusters with oxygen defects and high proportion of Co2þ for oxygen evolution reaction; Journal of Alloys and Compounds, Published by Elsevier B.V., 2019.

**Figure 4:**
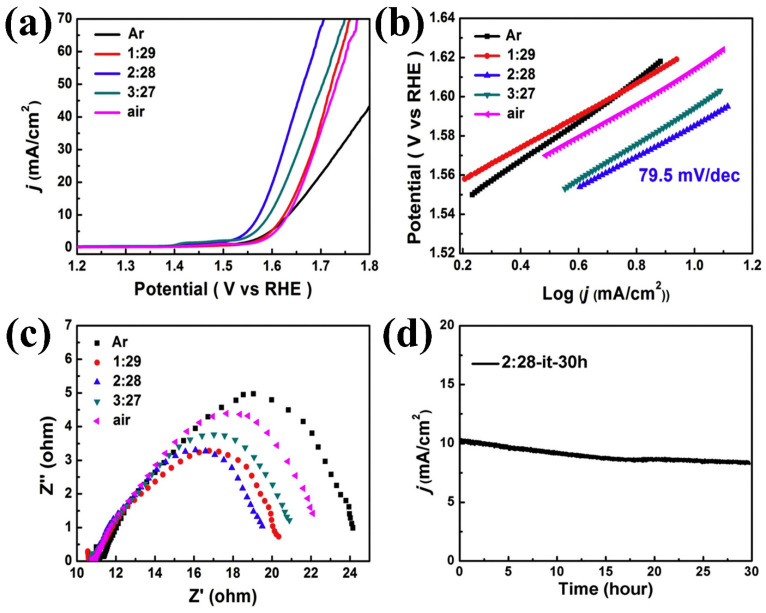
Oxygen evolution reaction (OER) performance under varying O_2_ and Ar flow rates: (**a**) Linear sweep voltammetry (LSV) curves, (**b**) Tafel plots, (**c**) electrochemical impedance spectroscopy measurements, and (**d**) stability test results of the optimum electrocatalyst over 30 h. Reproduced with permission from Zhu et al. [12], Special atmosphere annealed Co_3_O_4_ porous nanoclusters with oxygen defects and high proportion of Co2þ for oxygen evolution reaction; Journal of Alloys and Compounds, Published by Elsevier B.V., 2019.

Zhu et al. [12] synthesized nanoclusters comprising nanowires and nanosheets, targeting the optimization of OER activity by creating oxygen vacancies through the manipulation of the Co^2+^/Co^3+^ ion ratio. This ratio was adjusted via heat treatment under controlled O_2_ and Ar gas flows. A sample treated with an O_2_:Ar ratio of 2:28 demonstrated an overpotential of 343 mV and a Tafel slope of 79.5 mV/dec in 0.1 M KOH. X-ray photoelectron spectroscopy (XPS) analysis revealed that this sample exhibited a higher Co^2+^/Co^3+^ atomic ratio (1.26) compared to the air-annealed sample (1.00), confirming the formation of surface oxygen vacancies. The deconvoluted Co2p and O1s XPS spectra of both samples are presented in Figure 3a–d. Results derived from various analyses of overall OER activities, namely linear sweep voltammetry (LSV), Tafel plot analysis, electrochemical impedance spectroscopy, and 30-h stability tests for prominent samples, are illustrated in Figure 4a–d, respectively.

### 2.2. Chemical Stability in Alkaline Electrolytes

The chemical stability of Co_3_O_4_ in alkaline electrolytes is crucial for its effectiveness in long-term water-splitting applications. Alkaline electrolytes are commonly employed in water-splitting reactions because they mitigate corrosion and enable the use of a broader range of catalysts. Furthermore, compared to acidic environments, alkaline conditions present lower overpotentials for the OER and HER, resulting in more energy-efficient processes [48,49,50]. Alkaline electrolytes, such as potassium hydroxide (KOH), further facilitate the use of non-noble metal catalysts such as Co_3_O_4_, which are more abundant and cost-effective than precious metals such as iridium or platinum. The stability of Co_3_O_4_ in alkaline environments is essential for preserving its structural integrity and catalytic activity over extended water-splitting reactions. This chemical resilience of Co_3_O_4_ prevents surface degradation, thereby ensuring durability and reducing the frequency of catalyst replacement [51,52]. In particular, the spinel structure of Co_3_O_4_ enhances its resistance to corrosion during the OER, particularly in high-pH alkaline electrolytes where other metal oxides often degrade. This inherent corrosion resistance is a notable advantage for long-term, cost-effective applications, as it minimizes material loss and reduces the need for additional protective measures.

Moreover, as outlined in the previous section, the stability of Co_3_O_4_ in alkaline solutions is also influenced by its specific crystallographic facets. Chen et al. [53] examined the facet-dependent stability and OER performance of Co_3_O_4_, demonstrating that the octahedral facet with a (111) crystal plane is more stable than the cubic surface with a (100) plane. Kim et al. [54] investigated the effect of annealing temperature on Co_3_O_4_ nanoparticles synthesized through thermal metal–organic deposition on carbon fibers. Their findings revealed that Co_3_O_4_ nanoparticles annealed at 700 °C exhibited excellent stability, with an overpotential shift of only 1.86 mV (from 370 to 377 mV) after 5000 cyclic voltammetry (CV) sweep cycles, and maintained steady performance during a 10-h chrono-current test at 10 mA.

While the stability of Co_3_O_4_ in acidic environments remains limited, it can be improved through the meticulous selection of support materials, adjustments to thickness and crystallinity, and appropriate doping. Pascuzzi et al. [55] explored the effects of different substrates and Li-doping on Co_3_O_4_ stability in H_2_SO_4_, reporting that fluorine-doped-tin-oxide-supported Co_3_O_4_ films exhibited high stability over 16 h. Although Li-doping slightly reduced stability, it enhanced OER activity by lowering the potential required to reach a practical current density of 10 mA cm^−2^. The resilience of Co_3_O_4_ demonstrated across these studies highlights its potential for large-scale hydrogen production through water electrolysis, contributing to the advancement of sustainable energy systems.

### 2.3. Structural and Morphological Flexibility

**Figure 5:**
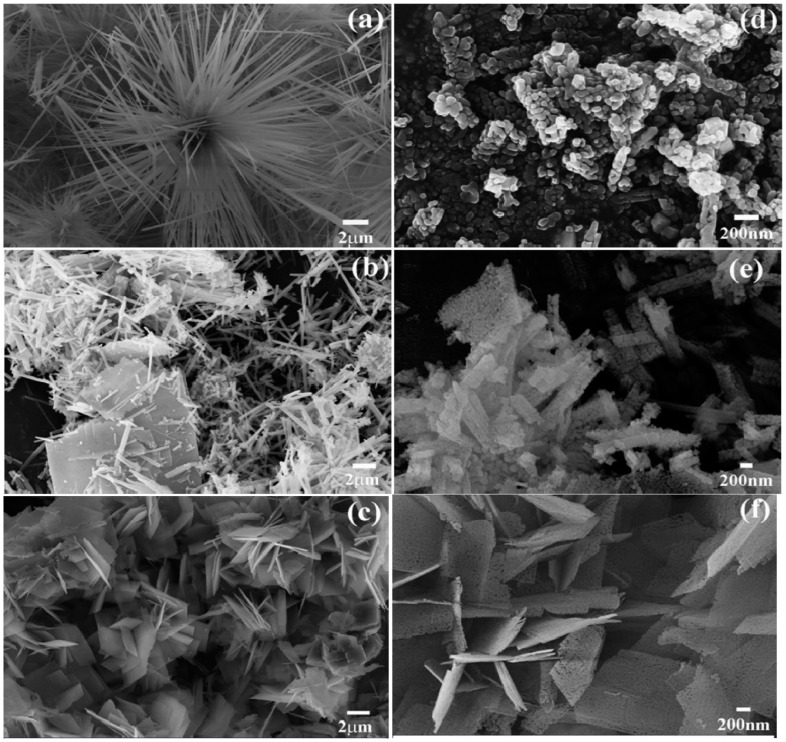
Impact of urea concentration and annealing on the structural evolution of Co_3_O_4_: (**a**–**c**) SEM images of Co_3_O_4_ samples prepared at different urea concentrations, and (**d**–**f**) SEM images of the corresponding samples after annealing at 400 °C. Reproduced with permission from Zhang et al. [13], Morphology-controlled fabrication of Co_3_O_4_ nanostructures and their comparative catalytic activity for oxygen evolution reaction; Journal of Alloys and Compounds, Published by Elsevier B.V., 2016.

**Figure 6:**
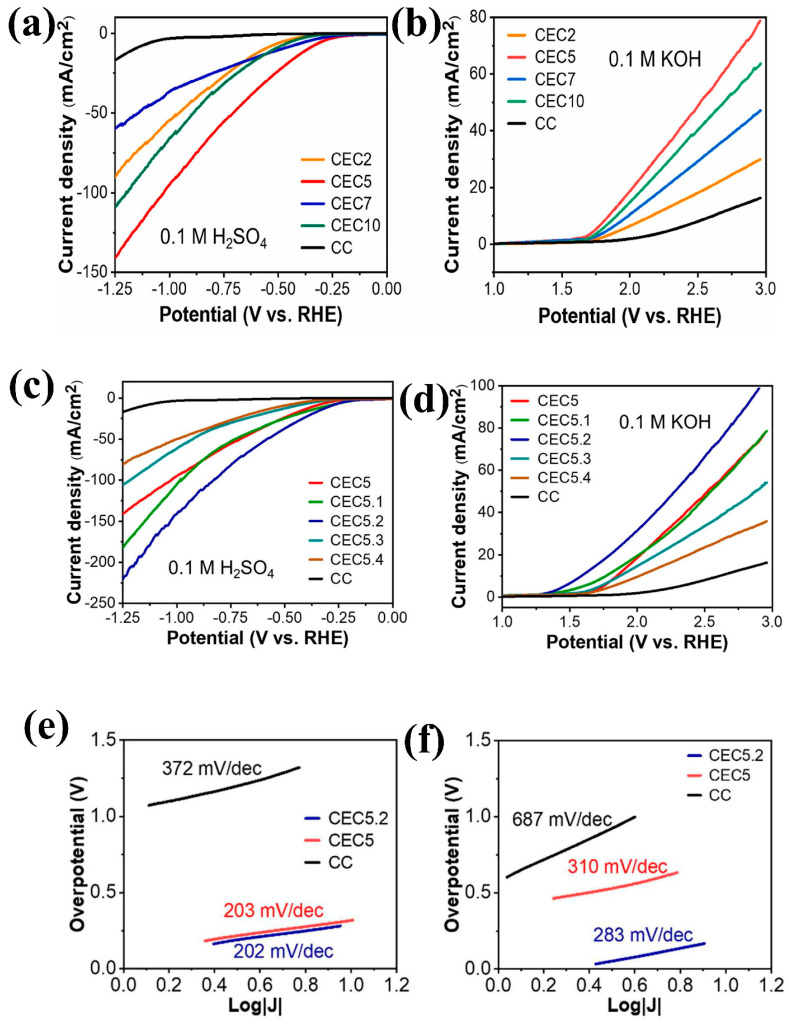
Hydrogen evolution reaction (HER) and oxygen evolution reaction (OER) catalytic performance of Co_3_O_4_ electrodeposited over different deposition durations and at different ethylene glycol-to-water (EG:W) ratios: (**a**) LSV curve of Co_3_O_4_ deposited over different deposition durations for the HER, (**b**) LSV curve of Co_3_O_4_ deposited over different deposition durations for the OER, (**c**) LSV curve of Co_3_O_4_ deposited at different EG:W ratios for the HER, (**d**) LSV of Co_3_O_4_ deposited at different EG:W ratios for the OER, (**e**) Tafel plots for the HER, and (**f**) Tafel plots for the OER. Reproduced with permission from La et al. [56], Development of Co_3_O_4_ nanomaterials on flexible carbon cloth substrates for hydrogen and oxygen evolution reactions; International Journal of Hydrogen Energy, Published by Elsevier Ltd., 2023.

Co_3_O_4_ can be synthesized with various nanostructures, including thin films, flakes, wires, and particles, which significantly enhance its catalytic efficiency for water splitting. These nanostructures increase the active surface area, facilitate electron and ion transport, improve catalytic performance, and provide greater stability under harsh conditions. Thus, the morphological flexibility of Co_3_O_4_ enables the optimization of its catalytic properties through tailored designs, making it highly effective and adaptable for water splitting and other renewable energy applications [56,57,58]. Zhang et al. [13] tuned the morphology of Co_3_O_4_ through a two-step process involving microwave–hydrothermal synthesis followed by post-calcination and examined the OER performance of the resulting structures. By adjusting the urea concentration in the solution, they synthesized various morphologies, namely nanorods, nanosheets, and their combinations. Annealing at 400 °C disassembled the nanorods into loosely aggregated nanoparticles, while the nanosheets developed a porous structure with a uniform pore distribution. SEM images of the samples recorded before and after annealing are presented in Figure 5a–f. Among the tested morphologies, the porous nanosheets demonstrated the best OER performance, achieving the lowest overpotential of 399 mV at 0.5 mA cm^−2^. This superior performance of the porous nanosheets can be attributed to their high surface area (24.80 m^2^g^−1^), which is greater than those of the other two samples. La et al. [56] utilized a straightforward electrodeposition strategy to grow well-defined Co_3_O_4_ crystallites on carbon fibers. They observed variations in the crystallite distribution and structural changes as the electrodeposition time increased from 2 to 10 min. The optimal crystallite distribution was achieved at a deposition time of 5 min, resulting in superior HER and OER performance in acidic (H_2_SO_4_) and alkaline (KOH) media, respectively. The electrode with Co_3_O_4_ crystallites deposited over 5 min exhibited overpotentials of 365 mV for the HER and 614 mV for the OER. Variations in the solvent ratio of ethylene glycol to water were also explored to form mesoporous micro-flakes uniformly distributed on carbon fibers. A 2:3 volume ratio of ethylene glycol to water yielded micro-flakes achieving the lowest overpotentials of 291 mV for the HER in H_2_SO_4_ and 357 mV for the OER in KOH. Figure 6a–f illustrates the HER and OER activities of Co_3_O_4_ samples deposited onto carbon fibers over varying durations and at different solution concentrations. Notably, the abovementioned electrode maintained high catalytic activity even after 30 h of chronopotentiometry at 10 mA cm^−2^ in an alkaline medium. Du et al. [21] synthesized nanomesh, nanorod, and nanowire structures of Co_3_O_4_ using varying cobalt precursors (nitrate, acetate, and chloride, respectively) and evaluated their OER activity in KOH. Among these configurations, the nanowire array exhibited superior OER performance, achieving an overpotential of 394 mV at 20 mA cm^−2^ compared to 425 mV for the nanomesh and 434 mV for the nanorod electrodes. Li et al. [57] employed a binder-free hydrothermal method to synthesize a 3D array of urchin-like Co_3_O_4_ spheres on Ni foam, which demonstrated excellent bifunctional catalytic performance for both the OER and HER. A polarization curve analysis at 2 mV s^−1^ revealed an onset potential of 1.46 V and an overpotential of 230 mV for the OER, along with an onset potential of −130 mV and an overpotential of −225 mV for the HER. Moreover, the catalyst exhibited long-term stability in both processes when tested at various current densities. The mesoporous structure of the catalyst, featuring numerous active sites and reduced charge transfer resistance, enhanced its bifunctional catalytic performance compared to that of conventional Co_3_O_4_ nanoparticles. Overall, tailored designs of Co_3_O_4_ allow fine-tuning of porosity, conductivity, and surface chemistry, delivering enhanced efficiency and stability for practical water-splitting systems.

### 2.4. Magnetic and Electronic Properties

**Figure 7:**
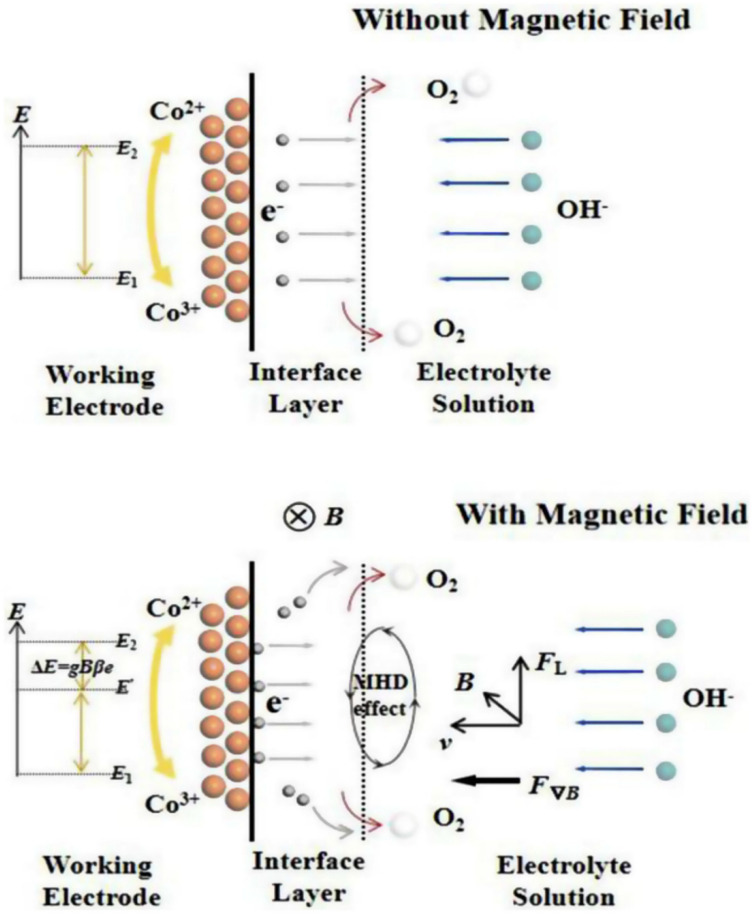
Mechanism illustrating the transition states of Co^2+^/Co^3+^ during the OER in a Co_3_O_4_ sample, comparing conditions without a magnetic field and under the influence of a magnetic field. Reproduced with permission from Li et al. [59], Magnetic field enhancing electrocatalysis of Co_3_O_4_/NF for oxygen evolution reaction; Journal of Power Sources, Published by Elsevier B.V., 2019.

The distinctive electronic and magnetic properties of Co_3_O_4_, attributed to its spinel structure, enhanced its performance as a water-splitting catalyst. Its semiconducting behavior facilitates efficient charge separation and transport, reducing recombination losses and improving overall catalytic efficiency. Additionally, the spinel structure of Co_3_O_4_ promotes enhanced electron transfer, making it highly effective for the OER and HER during water splitting. Co_3_O_4_ also displays intriguing magnetic properties owing to the presence of Co ions in various oxidation states. While Co_3_O_4_ is antiferromagnetic at low temperatures, this magnetic ordering can influence electron spin states, potentially improving the kinetics of water-splitting reactions. Li et al. [59] investigated the impact of a magnetic field on the OER performance of 3D urchin-like Co_3_O_4_ structures comprising nanoneedle bundles. Their findings revealed that as the magnetic field strengthened, the potential required to achieve a current density of 20 mA cm^−2^ dropped, with the highest OER activity observed when the magnetic field was perpendicular to the electric field. This effect arises because the magnetic field raises the spin energy states of Co^2+^/Co^3+^ electrons, increasing the activation energy for charge transfer and facilitating the catalytic reaction. Overall, the magnetic field influences redox electron transfer kinetics by raising electron energy levels, which accelerates the electron transfer process, as described by classical transition state theory. With increasing magnetic field strength, the energy differential (ΔE) grows, reducing the overpotential. The mechanism underlying the OER activity of Co_3_O_4_ with and without a magnetic field, based on this theory, is presented in Figure 7.

Similar to a magnetic field, an electric field can also enhance overall electrocatalytic performance. Li et al. [60] investigated the effects of an electric field on an Ni–Co_3_O_4_ composite thin film. Their findings revealed that applying an electric field substantially reduced the resistance of Ni–Co_3_O_4_, thereby enhancing its HER, OER, and electrolyzer performance to levels competitive with noble metals such as Pt/C and RuO_2_. In the low-resistance state, the film required overpotentials of only 93 mV and 311 mV to achieve a current density of 10 mA cm^−2^ during the HER and OER, respectively. This study highlights that reducing film resistance improves conductivity and enhances bifunctional catalytic activities.

## 3. Co_3_O_4_-Based Composite Electrocatalysts for the HER and OER

While Co_3_O_4_ exhibits several advantageous properties that establish its potential as a promising electrocatalyst for hydrogen generation, its standalone performance in large-scale water splitting remains inadequate. To enhance its electrocatalytic capabilities, Co_3_O_4_ must be combined with other high-performing materials. Effective options include carbon-based derivatives, other metal oxides, sulfides, materials derived from MOFs, and noble metals. This section examines recent advancements in Co_3_O_4_-based composite electrocatalysts for the HER and OER.

### 3.1. Composites of Co_3_O_4_ with Carbon Derivatives

Among the various strategies explored to date, integrating Co_3_O_4_ with carbon-based materials—such as graphene, activated carbon, carbon nanotubes (CNTs), carbon quantum dots, and graphitic carbon nitride (g-C_3_N_4_)—has proven effective. This is because carbon-based materials offer excellent electrical conductivity and a large surface area, which enhance charge transfer efficiency between the catalyst and the electrolyte [26,29,56,61,62,63,64,65,66,67,68,69,70,71,72]. Furthermore, combining Co_3_O_4_ with carbon-based materials mitigates Co_3_O_4_ nanoparticle aggregation, ensuring a uniform distribution of active sites [64,65,66]. Additionally, these materials enhance the mechanical stability of the composites, improving durability under long-term electrocatalytic conditions.

**Figure 8:**
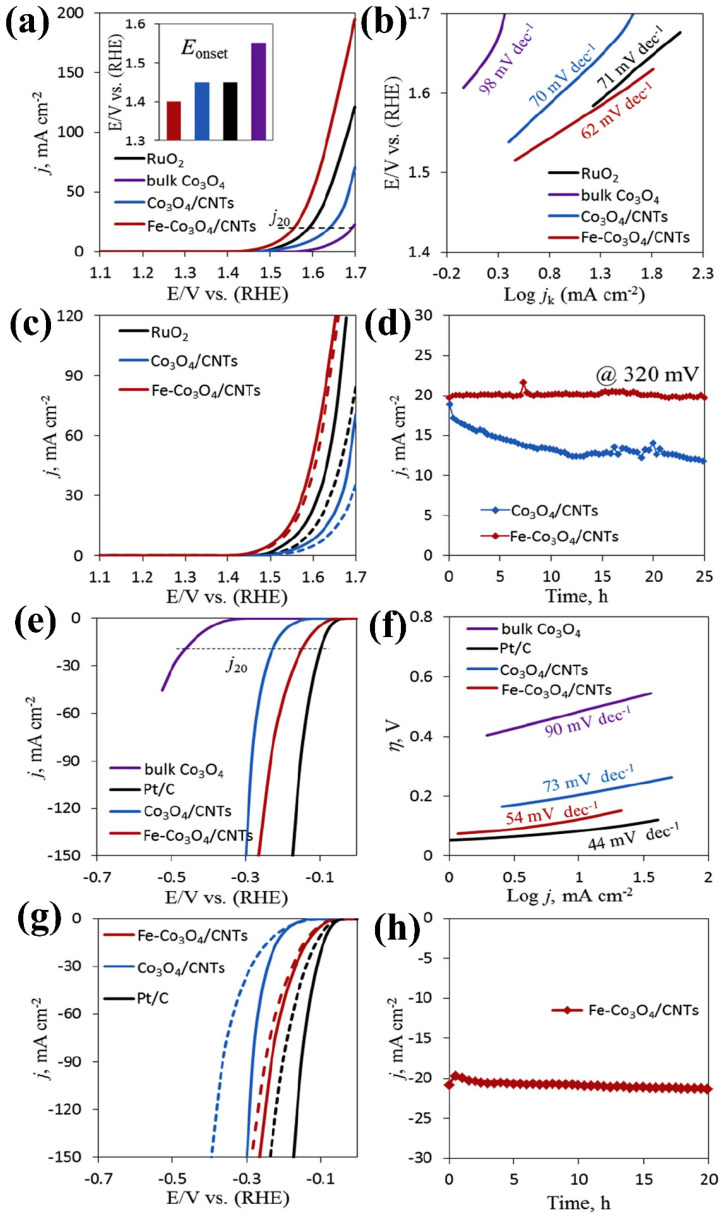
Comparative analysis of the OER and HER performance of Co_3_O_4_-based composites: (**a**) LSV curves depicting OER activity, (**b**) Tafel plots derived from OER measurements, (**c**) LSV curves before and after 1000 cycles, (**d**) OER stability test of two prominent catalysts, (**e**) LSV curves depicting HER activity, (**f**) Tafel plots derived from HER measurements, (**g**) LSV before and after 1000 cycles, and (**h**) HER stability test of prominent catalysts. Reproduced with permission from Begum, H., and Jeon, S. [61]. Highly efficient and stable bifunctional electrocatalyst for water splitting on Fe-Co_3_O_4_/carbon nanotubes; International Journal of Hydrogen Energy, Published by Elsevier Ltd., 2018.

Begum and Jeon [61] studied the HER and OER performance of a Co_3_O_4_ composite with multi-walled carbon nanotubes (MWCNTs). They also developed a core–shell Fe–Co_3_O_4_ structure combined with MWCNTs, which exhibited exceptional OER performance. For instance, this composite achieved an overpotential of 300 mV, outperforming the noble metal electrode RuO_2_, which presents an overpotential of 340 mV. The Fe–Co_3_O_4_–MWCNT composite also demonstrated superior HER activity, shifting the polarization curve closer to zero compared to the Co_3_O_4_ and Co_3_O_4_–MWCNT composites. The HER overpotentials of Fe–Co_3_O_4_–MWCNT, Co_3_O_4_–MWCNT, and Co_3_O_4_ were 120 mV, 180 mV, and 480 mV, respectively. Additionally, the electrocatalyst exhibited excellent stability over 20 h during both the OER and HER, making it suitable for sustained hydrogen production. Figure 8a–h illustrates the OER and HER activities of the Co_3_O_4_, Co_3_O_4_–MWCNT, and Fe–Co_3_O_4_–MWCNT electrocatalysts. Mohana et al. [62] evaluated the bifunctional capabilities (HER and OER) of the Co_3_O_4_–g-C_3_N_4_ composite. This catalyst achieved overpotentials of 170 mV and 151 mV for the OER and HER, respectively, at a current density of 20 mA cm^−2^, along with corresponding Tafel slopes of 188 mV dec^−1^ and 176 mV dec^−1^. Furthermore, the composite exhibited excellent stability, demonstrating only 26% and 40% degradation in OER and HER performance, respectively.

Yan et al. [63] applied CH_4_ plasma treatment to introduce intrinsic defects into Co_3_O_4_ nanosheets and incorporate atomic-level carbon doping. This defect engineering, combined with the formation of Co–C bonds, synergistically improved the HER and OER activities of the Co_3_O_4_ nanosheets compared to those of the untreated Co_3_O_4_ nanosheets. Notably, even a minimal addition of Co_3_O_4_ nanoparticles can substantially boost the HER and OER performance of materials. For instance, Jayaseelan et al. [64] developed a polypyrrole–MWCNT (Ppy-carbon) composite and observed that incorporating 5 wt% Co_3_O_4_ nanoparticles (8–10 nm size) enhanced its catalytic activity for both the HER and OER. However, increasing the Co_3_O_4_ content to 20 wt% compromised its bifunctional performance.

Abd-Elrahim and Chun [70] created a hybrid composite of nanostructured Co_3_O_4_ and graphene and tested its HER and OER activities. Their findings indicated that the composite containing 25 wt% Co_3_O_4_ demonstrated excellent performance for the OER, while the composite containing 50 wt% Co_3_O_4_ demonstrated superior HER activity. Experimental studies are frequently complemented by theoretical analyses, which provide a reliable and detailed understanding of the factors that enhance the overall electrocatalytic performance of a system. For instance, in the oxygen evolution reaction (OER), the surface adsorption of intermediates such as *OH, *OOH, and *O plays a crucial role in determining the desired OER activity. Similarly, catalysts that exhibit strong adsorption of the H* intermediate are generally more favorable for the hydrogen evolution reaction (HER).

A recent piece of research by Ahmed et al. [65] demonstrated that incorporating Co_3_O_4_ into the WO_3_/C system reduces the conversion of *OH to O, a key obstacle in achieving lower potential values for OER activity. Additionally, the introduction of Co_3_O_4_ optimizes the adsorption of H on the surface, thereby enhancing the HER activity of the Co_3_O_4_/WO_3_/C system. In their study, they developed a ternary system with Co_3_O_4_/WO_3_/C featuring a rod-shaped morphology, in which the activated carbon was derived from the cellulose waste (sugar cane bagasse). Such a composite system exhibits an overpotential of 229 and 123 mV during the OER and HER activity when tested in alkaline and acidic medium respectively with excellent stability for 24 h in both cases. However, the OER performance degraded when the electrolyte medium was changed from alkaline (1 M KOH) to acidic (0.5 M H_2_SO_4_). The same catalysts in an acidic medium exhibit an overpotential of 295 mV in an acidic medium. In another study, Ahmed et al. [66] synthesized a composite of Co_3_O_4_ nanorods with MoO_3_ and the conductive carbon derivative g-C_3_N_4_. The composite, Co_3_O_4_/MoO_3_/g-C_3_N_4_, was prepared using a three-step hydrothermal process. This material demonstrated a low overpotential of 206 mV for OER in an alkaline electrolyte and a comparable overpotential of 125 mV for HER in an acidic medium, with consistent durability over 24 h. Table 1 provides a comparative summary of the HER and OER performance of Co_3_O_4_ and its composites with carbon derivatives from various studies.

### 3.2. MOF-Derived Composites of Co_3_O_4_

Apart from carbon derivatives, integrating Co_3_O_4_ into MOFs or covalent–organic frameworks (COFs), which are highly porous materials with precise structural organization and large surface areas, presents another promising strategy for enhancing catalytic performance. The adjustable porosity of MOFs facilitates efficient mass transfer of reactants and products, thereby improving reaction kinetics. Additionally, the metal nodes within MOFs contribute to the catalytic activity of Co_3_O_4_. MOF-derived Co_3_O_4_ composites also exhibit enhanced thermal and chemical stability, making them well-suited for electrocatalytic water splitting under challenging conditions. These combined attributes establish Co_3_O_4_–MOF/COF composites as advanced materials for sustainable energy applications [23,30,73,74,75,76,77,78,79,80].

**Figure 9:**
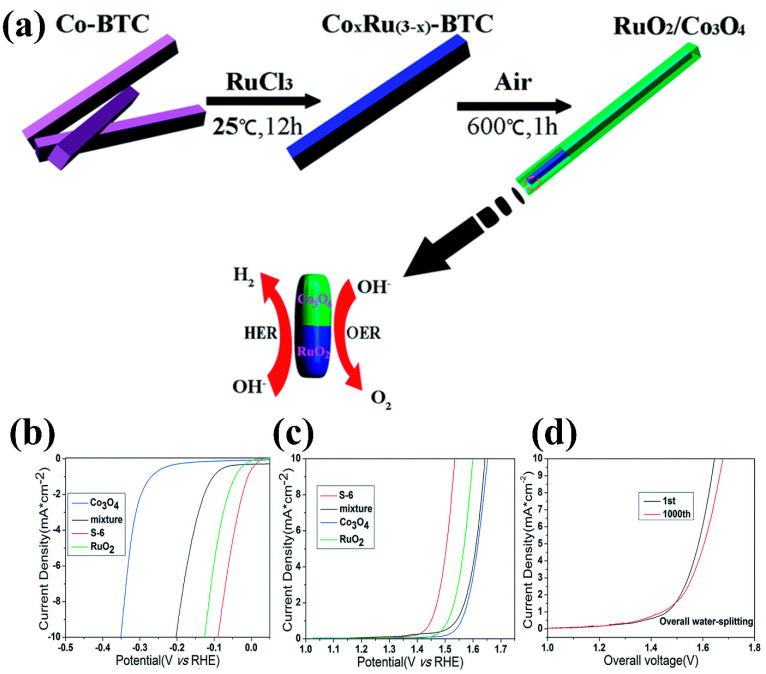
(**a**) Design strategy of the RuO_2_/Co_3_O_4_ composite utilizing a metal–organic framework (MOF), (**b**) HER activity with and without RuO_2_, (**c**) OER activity with and without RuO_2_, and (**d**) LSV curves of the RuO_2_/Co_3_O_4_ǀǀ RuO_2_/Co_3_O_4_ electrolyzer cell during the first and after 1000 cycles. Reproduced with permission from Liu et al. [30], MOF-derived RuO_2_/Co_3_O_4_ heterojunctions as highly efficient bifunctional electrocatalysts for HER and OER in alkaline solutions; RSC Advances, Published by, The Royal Society of Chemistry, 2017.

For instance, Liu et al. [30] developed an RuO_2_–Co_3_O_4_ heterojunction derived from an MOF. The Co–Ru precursor retained its rectangular bar-like structure even after annealing at 600 °C. The synthesis process for the RuO_2_–Co_3_O_4_ heterostructure is depicted in Figure 9a. The HER and OER activities of RuO_2_–Co_3_O_4_ samples with varying Co:Ru molar ratios were evaluated. The catalyst with a 6:1 Co: Ru ratio exhibited superior performance in the OER, HER, and overall water splitting, as depicted in Figure 9b–d. Remarkably, despite its low precious metal content, the RuO_2_–Co_3_O_4_ composite exhibited substantially improved electrocatalytic activity compared to both pure Co_3_O_4_ and commercial RuO_2_. The catalyst with a 6:1 Co: Ru ratio achieved overpotentials of 305 mV for the OER and 89 mV for the HER, maintaining stability over 10,000 cycles. This enhancement is attributed to the heterojunction structure of the composite, which creates highly active catalytic sites at the RuO_2_–Co_3_O_4_ interface. MOFs offer a versatile platform for creating hybrid structures by combining materials such as metal oxides and conductive polymers. The synergy between these materials enhances the electrocatalytic performance of the electrodes.

**Figure 10:**
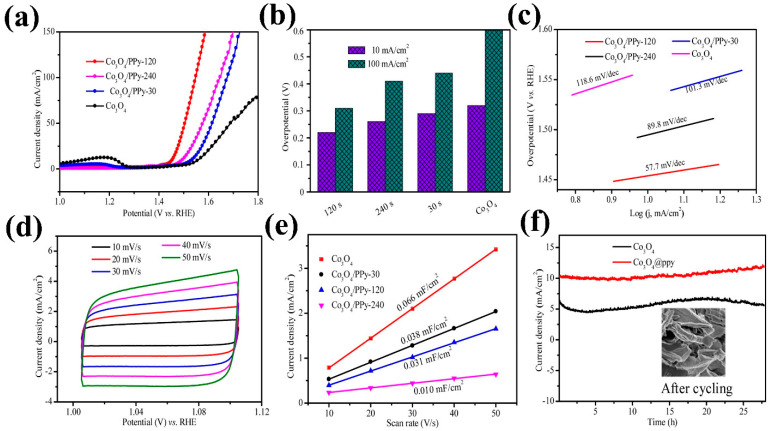
OER activity of the Co_3_O_4_/polypyrrole (Ppy) composite: (**a**) LSV curves of the Co_3_O_4_/Ppy composites subjected to varying Ppy deposition times, (**b**) estimated overpotentials for OER activity at current densities of 10 and 100 mA cm^−2^, (**c**) Tafel plots for OER activity, (**d**) CV curves at different scan rates, (**e**) estimated electrochemical surface area (ECSA), and (**f**) OER stability of the Co_3_O_4_ and Co_3_O_4_/Ppy composite (inset: SEM image after the stability test). Reproduced with permission from Tong et al. [75], Metal-organic framework derived Co_3_O_4_/PPy bifunctional electrocatalysts for efficient overall water splitting; Chinese Chemical Letters, Published by, Elsevier B.V., 2020.

**Figure 11:**
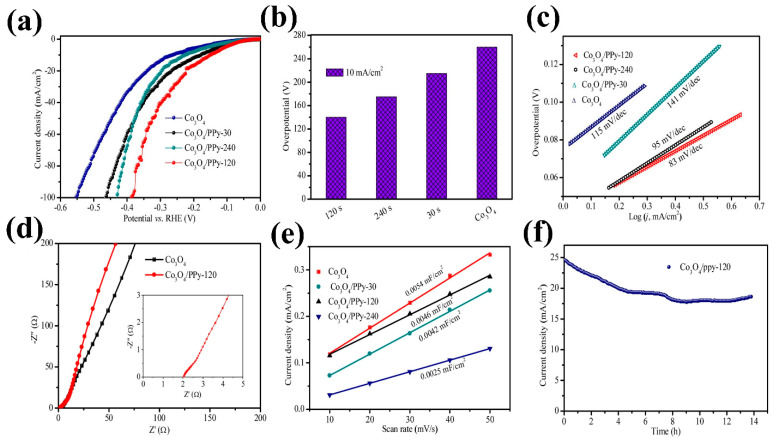
HER activity of the Co_3_O_4_/Ppy composite: (**a**) LSV curves of the Co_3_O_4_/Ppy composites subjected to varying Ppy deposition times, (**b**) estimated overpotentials for HER activity at a current density of 10 mA cm^−2^, (**c**) Tafel plots for HER activity, (**d**) electrochemical impedance spectroscopy spectra of Co_3_O_4_ and the Co_3_O_4_/Ppy composite, (**e**) estimated ECSA, and (**f**) HER stability of the Co_3_O_4_/Ppy composite. Reproduced with permission from Tong et al. [75], Metal-organic framework derived Co_3_O_4_/PPy bifunctional electrocatalysts for efficient overall water splitting; Chinese Chemical Letters, Published by, Elsevier B.V., 2020.

**Figure 12:**
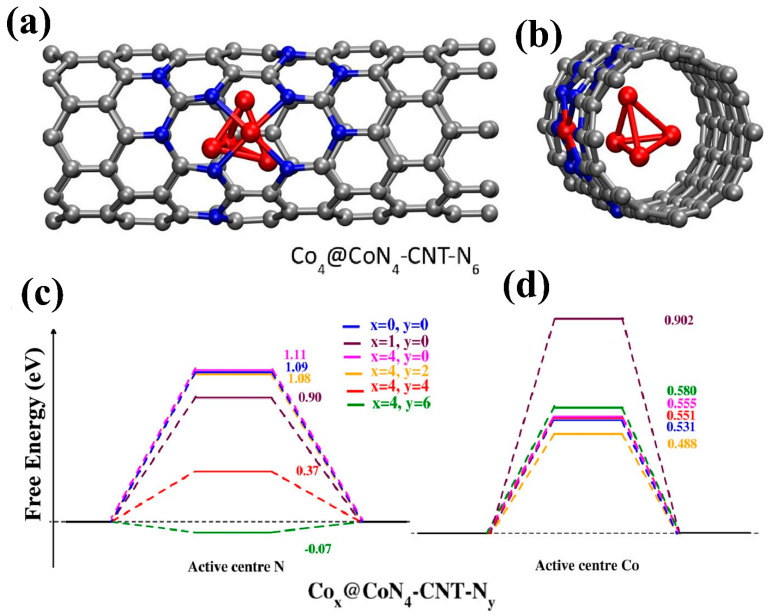
Theoretical model for the composite with prominent HER activity (Co_4_@CoN_4_–CNT–N_6_ composite): (**a**) front view, (**b**) side view, (**c**) free energy plot of H-adsorption at the N-center, and (**d**) H-adsorption at the Co-center. Reproduced with permission from Singh et al. [76], MOF Derived Co3O4@Co/NCNT nanocomposite for electrochemical hydrogen evolution, flexible zinc-air batteries, and overall water splitting; Inorganic Chemistry, Published by American Chemical Society, 2020.

Tong et al. [75] developed a Co_3_O_4_–Ppy hybrid, where Ppy was electrodeposited over varying durations. The bifunctional catalytic activity of this hybrid material revealed that Ppy deposition time influences polarization behavior in both the OER and HER. As illustrated in Figure 10a–f, the hybrid electrode subjected to 120 s of Ppy deposition exhibited stability over 25 h and achieved optimal overpotentials of 220 mV for OER and 140 mV for HER. The HER performance of the hybrid electrode is depicted in Figure 11a–f. Sing et al. [76] employed an MOF to create a semiconducting metallic core–shell structure, Co_3_O_4_@Co, combined with nitrogen-doped carbon nanotubes. The trifunctional electrocatalytic activities (HER, OER, and oxygen reduction reaction (ORR)) of this composite electrocatalyst were tested in an acidic H_2_SO_4_ environment. Interestingly, the composite demonstrated promising performance as a flexible electrode for zinc–air batteries. The MOF-derived electrode achieved a low overpotential of 171 mV for the HER. The theoretical analysis, presented in Figure 12a–d, underscores the role of nitrogen concentration in enhancing the performance of this composite by providing active sites for H^+^ adsorption. Generally, various factors compromise the speed and efficiency of synthetic processes in chemical and material sciences.

One promising strategy to address this is the MOF@MOF approach, which enables the rapid formation of hybrid composites of metal oxides and carbon. For instance, Dung et al. [74] adopted a dual MOF strategy to synthesize an Ni/Co/Co_3_O_4_@C composite, using a microwave technique to form Ni–MOF@Co–MOF within minutes. Subsequently, laser scribing was employed to hybridize the Ni/Co/Co_3_O_4_ nanoparticles, encasing them in graphitic carbon. The resulting composite exhibited bifunctional electrocatalytic performance, with overpotentials of 246 mV for the OER and 143 mV for the HER. Furthermore, the electrolyzer, tested in a two-terminal system, maintained 91.6% efficiency after 24 h of continuous operation.

**Figure 13:**
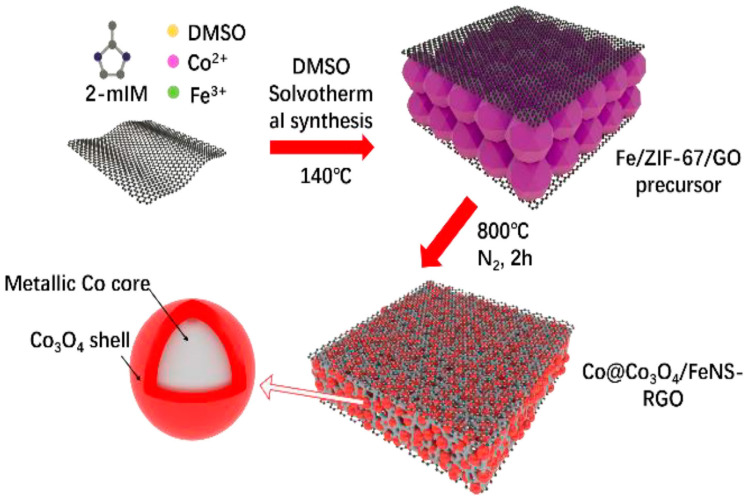
Schematic representation of the synthetic process of the Co@Co_3_O_4_/FeNS-rGo composite. Reproduced with permission from Zhu et al. [77], Zeolitic-imidazolate-framework-derived Co@Co_3_O_4_ embedded into iron, nitrogen, sulfur Co-doped reduced graphene oxide as efficient electrocatalysts for overall water splitting and zinc-air batteries; Electrochimica Acta, Published by Elsevier Ltd., 2019.

Zeolitic imidazolate frameworks (ZIFs) are a subclass of MOFs composed of imidazolate ligands and metal ions, typically cobalt or zinc. They feature variable pore configurations and a zeolite-like architecture, characterized by strong chemical and thermal stability. Their porous structure and large surface areas make them suitable for diverse applications, including gas storage, separation, catalysis, and electrochemical processes. Additionally, ZIFs can act as templates or precursors for synthesizing improved nanostructured materials, such as metal oxides or carbon composites, for energy-related applications like electrocatalysis [23,81]. For instance, Tang et al. [73] synthesized a ZIF@LDH precursor on Ni foam and conducted calcination treatments at varying temperatures to achieve complete dispersion of Co_3_O_4_ nanoparticles. When used as an electrocatalyst, this material achieved an HER overpotential of −106 mV at −10 mA cm^−2^ and an OER overpotential of 318 mV at 10 mA cm^−2^. Meanwhile, a two-electrode system using the above material as a monolithic catalyst for both the anode and cathode demonstrated a stable current density of 10 mA cm^−2^ with an applied voltage of 1.59 V for overall water splitting. Zhu et al. [77] utilized ZIF-67 to synthesize a Co@Co_3_O_4_/FeNS-RGO composite, as illustrated in Figure 13. This catalyst exhibited low overpotentials of 287 mV for the OER and 130 mV for the HER during ORR, OER, and HER activity tests.

**Figure 14:**
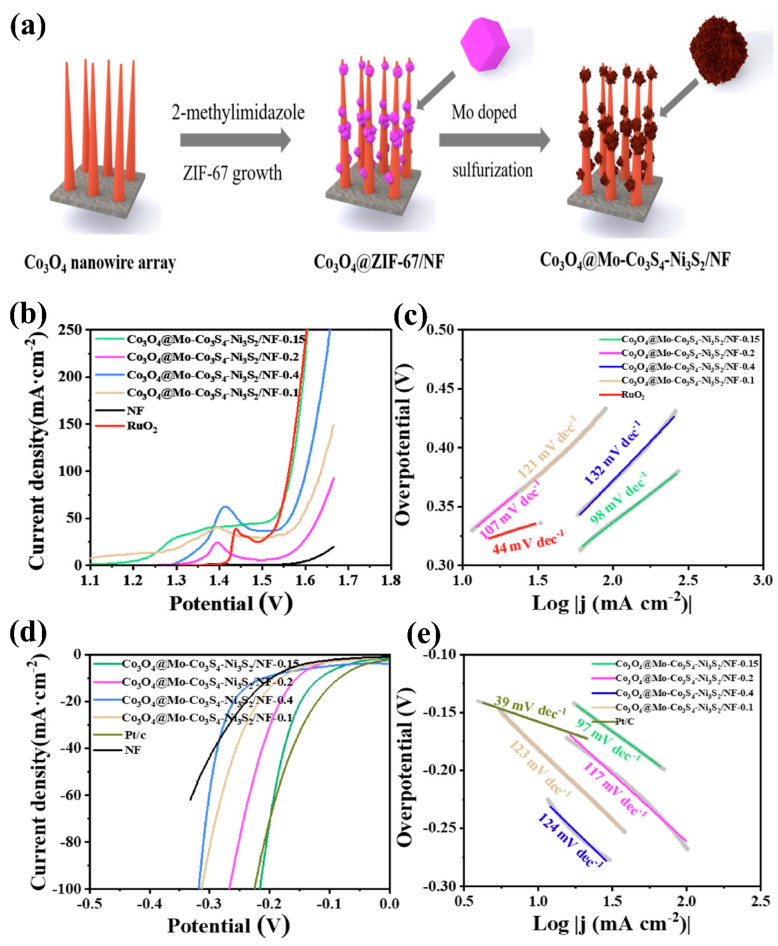
(**a**) Synthesis mechanism of Co_3_O_4_@Mo–Co_3_S_4_–Ni_3_S_2_/NF, (**b**,**c**) LSV curve depicting the OER activity and corresponding Tafel plots, and (**d**,**e**) LSV curve depicting the HER activity and corresponding Tafel plots. Reproduced with permission from Wu et al. [79], Metal-organic framework derived Co_3_O_4_@Mo-Co_3_S_4_-Ni_3_S_2_ heterostructure supported on Ni foam for overall water splitting; Electrochimica Acta, Published by Elsevier B. V., 2020.

Furthermore, a straightforward sulfurization process could transform ZIF-67-derived architectures into metal oxide–metal sulfide composites, further enhancing their electrocatalytic performance. For example, Wu et al. [79] synthesized an array of Co_3_O_4_ nanowires using ZIF-67 and produced Co_3_O_4_@Mo–Co_3_S_4_–Ni_3_S_2_-heterostructured catalysts on nickel foam. The synthesis process involved sulfurizing Co_3_O_4_ nanowires using ZIF-67 deposited on nickel foam, incorporating Mo doping to form the desired architecture. The fabrication steps for this architecture are depicted in Figure 14a. The resulting Co_3_O_4_@Mo–Co_3_S_4_–Ni_3_S_2_/NF heterostructure demonstrated low overpotentials of 295 mV for OER at 50 mA cm^−2^ and 116 mV for HER at 10 mA cm^−2^ under alkaline conditions. Additionally, the Co_3_O_4_@Mo–Co_3_S_4_–Ni_3_S_2_/NFǀǀCo_3_O_4_@Mo–Co_3_S_4_–Ni_3_S_2_/NF electrolyzer exhibited excellent catalytic efficiency for overall water splitting, achieving a current density of 10 mA cm^−2^ at a low voltage of 1.62 V. Meanwhile, Mo doping enhanced the performance of the heterostructure composite by creating additional positively charged centers, which facilitated electron transport and improved catalytic activity. The LSV curves depicting the OER and HER activities of the catalyst, along with corresponding Tafel plots, are illustrated in Figure 14b–e, demonstrating the influence of Mo concentration.

**Figure 15:**
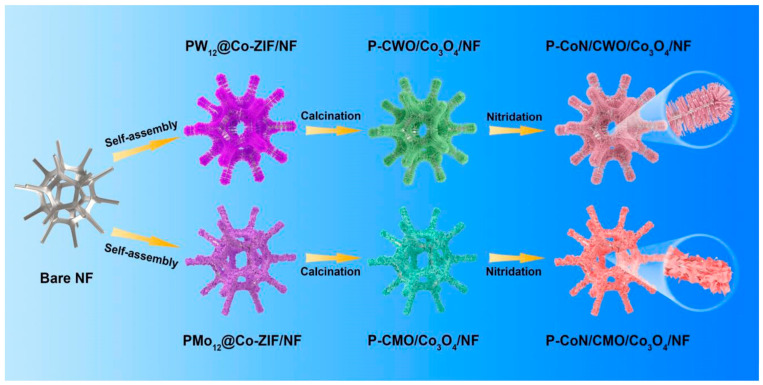
The synthesis process of P–CoN/CWO/Co_3_O_4_ and P–CoN/CMO/Co_3_O_4_ utilizing polyoxometalate-based metal-organic frameworks (POMOFs) and nitridation. Reproduced with permission from Ran et al. [83], P-doped Co-based nanoarray heterojunction with multi-interfaces for complementary HER/OER electrocatalysts towards high-efficiency overall water splitting in alkaline; International Journal of Hydrogen Energy, Published by Elsevier Ltd., 2024.

Another promising approach to designing systems with dual HER and OER functionalities is the use of polyoxometalate-based metal-organic frameworks (POMOFs). POMOFs are formed by integrating organic and inorganic components, each possessing unique physicochemical properties. This integration results in a system that combines multiple advantageous features, such as a larger surface area and higher porosity, which enhance redox capabilities and facilitate efficient electron transport during electrocatalytic processes [82]. Ran et al. [83] utilized polyoxometalate-based metal-organic frameworks (POMOFs) to develop an asymmetric electrolyzer featuring two distinct nanoarray heterojunctions with phosphorus (P) doping. Using a calcination-nitridation method, they fabricated P–CoN/CWO/Co_3_O_4_ (anode) and P–CoN/CMO/Co_3_O_4_ (cathode) nanoarray heterojunctions directly on nickel foam through an in-situ growth process. The fabrication process is illustrated in Figure 15. In an alkaline medium, the anode exhibited a low overpotential of 175 mV for oxygen evolution reaction (OER) and maintained stability for over 80 h during chronoamperometry testing at a current density of 10 mA cm^−2^. Similarly, the cathode demonstrated a minimal overpotential of 109 mV for hydrogen evolution reaction (HER) and showed excellent stability for more than 110 h. The electrolyzer, utilizing these asymmetric electrocatalysts, achieved a low overall overpotential of 1.55 V and remained stable for over 96 h. This study highlights the advantages of incorporating transition metal nitrides through a simple nitridation process, such as enhanced electrical conductivity and improved resistance to acidic and basic environments. Furthermore, P doping was found to be effective in tuning the chemical and structural properties, further boosting electrocatalytic performance. Table 2 compares the performance of various MOF-derived Co_3_O_4_ composite electrocatalysts reported previously.

### 3.3. Composites of Co_3_O_4_ with Other Materials

Beyond carbon materials and MOFs, transition metal hydroxides, oxides, sulfides, phosphides, and dichalcogenides have been investigated to enhance the catalytic performance of Co_3_O_4_-based systems. For instance, combining materials such as nickel oxide (NiO), nickel-cobalt oxide (NiCoO_2_), titanium oxide (TiO_2_) nickel–cobalt phosphate (NiCoP), iron oxide (Fe_2_O_3_), molybdenum disulfide (MoS_2_), cobalt selenide (CoSe_2_), cobalt sulfide (Co_3_S_4_), or heteroatoms like P with Co_3_O_4_ has been demonstrated to create heterojunctions that improve charge separation and reduce overpotentials for the HER and OER [17,18,19,20,26,27,60,84,85,86,87,88,89,90,91,92,93,94]. Additionally, incorporating trace amounts of noble metals such as Ru, Ag, and Au into heterojunctions or composites has been shown to further enhance the overall HER and OER electrocatalytic performance of Co_3_O_4_ [14,22,95,96,97,98]. This section discusses the performance of these composites.

**Figure 16:**
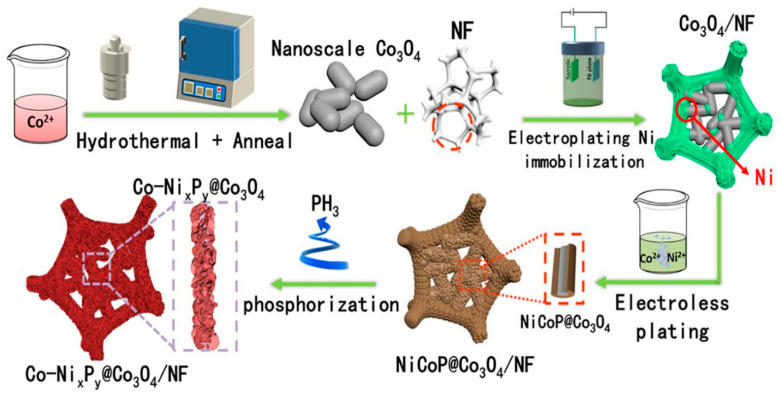
Synthesis process of the Co–Ni_x_P_y_@Co_3_O_4_/NF composite. Reproduced with permission from Lu et al. [17], Co-doped Ni_x_P_y_ loading on Co_3_O_4_ embedded in Ni foam as a hierarchically porous self-supported electrode for overall water splitting; Chemical Engineering Journal, Published by Elsevier B. V., 2021.

Transition metal phosphides (TMPs), which are abundant and cost-effective non-precious metal catalysts, have attracted increasing attention [35]. TMPs are widely used in systems such as fuel cells and lithium batteries, as well as in processes like photoelectrocatalysis. Leveraging the synergistic effects between metal species and TMPs is an effective approach for designing bifunctional catalysts with enhanced intrinsic activity. This involves the utilization of bimetallic and multimetallic TMPs, which further enhance the intrinsic activity of the catalyst. Lu et al. [17] synthesized a Co-doped nickel phosphide and Co_3_O_4_ composite on Ni foam. The fabrication of Co-Ni_x_P_y_@Co_3_O_4_/NF followed a three-step process: hydrothermal synthesis of Co_3_O_4_ nanoparticles, electrodeposition, and phosphating. This fabrication process is schematically illustrated in Figure 16. The Co–Ni_x_P_y_@Co_3_O_4_/NF electrode exhibited strong metal atom synergy, high conductivity, and a large surface area, which collectively enhanced its effectiveness for electrocatalytic processes. It achieved low overpotentials of 72 mV and 120 mV for the HER and OER at 10 mA cm^−2^, respectively. During overall water splitting, the self-supported electrode operated efficiently, requiring only 1.47 V to achieve a current density of 20 mA cm^−2^ in 1 M KOH, and maintained stability for 36 h. Notably, the ECSA of a catalyst plays a crucial role in its overall electrocatalytic performance, as a higher ECSA corresponds to an increased number of active sites. Transition metal oxides, such as Co_3_O_4_, provide ample active sites, thus enhancing ECSA and catalytic activity.

Tian et al. [35] developed a heterojunction catalyst, CoP/Co_3_O_4_, with a nanowire morphology grown on titanium mesh (TM). The synergistic integration of CoP and Co_3_O_4_ significantly enhanced the electrocatalytic performance of the CoP/Co_3_O_4_@TM system. In a 1 mol L^−1^ KOH solution, the catalyst demonstrated an overpotential of 257 mV at 20 mA cm^−2^ for the oxygen evolution reaction (OER) and 98 mV at 10 mA cm^−2^ for the hydrogen evolution reaction (HER).

The use of earth-abundant oxide materials is gaining importance in the development of electrocatalytic systems due to their stability in harsh environments, ease of production, low cost, and environmentally friendly properties. These characteristics make them highly suitable for large-scale industrial applications while reducing dependency on noble metals such as Ru, Ir, and Pt. Furthermore, growing heterostructures of these materials on three-dimensional conductive substrates is particularly advantageous, as these substrates enhance the accessibility of active sites and promote efficient charge transport across the electrocatalyst surface. Pan et al. [86] developed a nickel foam-supported NiCoO_2_@Co_3_O_4_ system comprising nanosheets of NiCoO_2_ and nanowire arrays of Co_3_O_4_ using a two-step hydrothermal method. The presence of oxygen vacancies and the *OOH intermediate contributed to its excellent OER and HER activities, achieving an overpotential of 79.9 mV for OER and 88.2 mV for HER under alkaline conditions, with stability exceeding 20 h. These results demonstrate its potential for overall water-splitting applications.

Atomic engineering has also proven effective in regulating the complex structures of metal oxides, enabling the fabrication of more stable electrocatalytic systems, even in acidic environments. For example, Wang et al. [87] synthesized three-dimensional nanofibers integrated with ultrafine Ni-Co_3_O_4_ particles using a simple electrospinning and calcination process. By optimizing the Ni doping concentration to 13.4%, the system achieved optimal bifunctional electrocatalytic activities in acidic media, delivering an overpotential of 74 mV for HER and 330 mV for OER. These findings highlight the potential of Ni-doped Co_3_O_4_-based systems for robust and efficient electrocatalysis. Mugheri et al. [91] synthesized a Co_3_O_4_–NiO composite using a straightforward two-step chemical synthesis method. The incorporation of NiO enhances conductivity and creates numerous active sites within the composite, leading to superior bifunctional activities compared to pristine NiO and Co_3_O_4_. During the HER, the composite electrocatalyst achieved a reduced dynamic potential, as indicated by its lower Tafel slope (61 mV dec^−1^) compared to those of Co_3_O_4_ (139 mV dec^−1^) a nd NiO (100 mV dec^−1^). Similarly, during the OER, the composite achieved an even lower Tafel slope of 101 mV dec^−1^, compared to 170 mV dec^−1^ for NiO and 287 mV dec^−1^ for Co_3_O_4_.

Incorporating heteroatoms into electrocatalysts as anions or cations is an effective strategy for modifying their electronic structure and enhancing electrocatalytic activity. This doping process alters the local electronic environment of the catalyst, improving charge distribution, conductivity, and active site availability depending on the specific heteroatom used [32,33,83,85,94]. By carefully selecting and optimizing the type and concentration of heteroatoms, researchers have developed highly efficient electrocatalysts with enhanced stability and performance for various energy conversion and storage applications. For instance, Duan et al. [32], used nitrogen (N) and sulfur (S) to fill the generated oxygen vacancies (Vo) in the Co_3_O_4_ lattice. In this work, they used in-situ etching of Co_3_O_4_ nanosheets using Ar-plasma these vacancies were further filled with heteroatoms N and S using thiourea through an in-situ process. Such a system of N/S/Vo-Co_3_O_4_ enables an overpotential of 181 mV during the HER process and 294 mV in the OER process.

Similarly, Xiao et al. [85] introduced phosphorus into a Co_3_O_4_ lattice rich in oxygen vacancies using the same method of etching and filling vacancies. However, he used to dope with P in vacancy-rich Co_3_O_4_. The incorporation of phosphorus filled these vacancies facilitated faster electron transport between Co2p states and enhanced bifunctional electrocatalytic activities. Additionally, P-doping improved the conductivity of pristine Co_3_O_4_. This heteroatom doping approach reduced the HER overpotential to 120 mV, which is 3–4 times lower than those of pristine Co_3_O_4_ (460 mV) and vacancy-rich Co_3_O_4_ (430 mV) at 10 mA cm^−2^. For the OER, the P-doped Co_3_O_4_ electrocatalyst achieved a current density of 10 mA cm^−2^ at 280 mV, compared to 340 mV for pristine Co_3_O_4_ and 330 mV for oxygen-vacancy-rich Co_3_O_4_. Although both works follow the same line for fabricating the prominent Co_3_O_4_-based system for electrocatalyst it is noted that choosing of heteroatom for doping is also more crucial in optimization of the performance.

Another promising approach to achieve optimal performance involves combining metal oxides with transition metal sulfides and/or transition metal dichalcogenides (TMDs). Wang et al. [40] fabricated a dual vacancy-rich heterojunction of Co_3_O_4_/Co_3_S_4_ using hydrothermal synthesis and soaking in sulfur precursor solution. Interestingly the electrolyzer based on such a heterojunction enables large stability over 200 h in a KOH solution. Meanwhile, this system also has a lower overpotential of 99 mV and only 26 mV in the OER and HER processes.

In the other instance, Balaji et al. [88] W-doped CoSe/Co_3_O_4_ heterojunction utilizing three steps. Fabrication of the WCo precursor is done at the first step followed by the selenization and oxidation take place to get the WCoSe/WCo_3_O_4_ heterojunction. The electrolysis cell developed using this system is also capable of delivering long-term stability of 100 h while the heterojunction delivers an overpotential of 175 mV during the OER process and 77 mV during HER. Very similar fashion Wang et al. [89] developed a heterojunction system comprising Co_3_S_4_/Co_3_O_4_ with the use of W-doping. It is noted that changing selenide to sulfur degrades the performance of the heterojunction, as this system enables overpotentials of 260 mV and 140 mV for OER and HER activities. Moreover, the stability of the overall cell is just 35 h below half of the noted for the system developed with selenide. Recently, Yu et al. [90] developed a Co_3_O_4_–MoSe_2_@C nanocomposite electrocatalyst and evaluated its bifunctional characteristics. This electrocatalyst demonstrated low overpotentials of 144 mV for the HER and 360 mV for the OER.

Incorporating a small amount of precious metals can enhance the electronic structure of Co_3_O_4_, improving its efficacy for both the HER and OER. For instance, Li et al. [14] developed a PtRu-Co_3_O_4_ electrocatalyst with an ultralow combined Pt and Ru content of only 0.23 mg cm^−2^. Fabricated using a simple electrodeposition technique, this system was employed as an electrolyzer in an acidic medium, demonstrating excellent performance and stability. It achieved voltages of 1.52 V and 1.63 V at current densities of 10 mA cm^−2^ and 100 mA cm^−2^, respectively. The cell exhibited remarkable durability, maintaining consistent performance for up to 100 h at 100 mA cm^−2^. Furthermore, it delivered a low overpotential of 143 mV for OER and 99 mV for HER at 10 mA cm^−2^, with stability sustained over 100 h for both processes.

Jiang et al. [34] demonstrated that an electrocatalyst fabricated without the use of a metal-organic framework (MOF) can outperform MOF-based systems in certain aspects. They developed a RuO_2_@Co_3_O_4_ electrocatalyst through a straightforward reduction reaction and calcination process, incorporating different amounts of Ru ions to optimize the system. Their study revealed that a Ru ratio of 1:6 yielded the best performance, comparable to the RuO_2_@Co_3_O_4_ heterojunction developed by Liu et al. [30], which utilized MOFs as a structural precursor. Interestingly, while the HER performance of both systems in an alkaline medium is similar, the electrocatalyst fabricated without MOFs exhibits superior OER activity, achieving a significantly lower overpotential of 152 mV compared to its MOF-based counterpart, which requires only half the potential to reach a current density of 10 mA cm^−2^. Furthermore, this non-MOF system delivers an overpotential of just 90 mV for HER in alkaline conditions. In acidic media, the RuO_2_@Co_3_O_4_ electrocatalyst maintains impressive performance, with overpotentials of 218 mV for OER and 33 mV for HER, further showcasing its versatility and robustness across different environments. These findings underline that MOFs, while advantageous for precise structural control, are not always necessary for achieving high-performance electrocatalysts. The simplified fabrication method employed by Jiang et al. reduces the complexity and cost associated with MOF-based approaches while delivering comparable or even superior performance. This opens new avenues for the development of cost-effective, scalable, and efficient electrocatalysts for water-splitting applications.

Feng et al. [96] developed a strawberry-like Co_3_O_4_–Ag structure using laser ablation. In this structure, Co_3_O_4_ facilitated water dissociation and optimized hydrogen adsorption on Ag by inducing tensile strain and reducing the coordination number. This mechanism resulted in a low HER overpotential of 51 mV. Furthermore, the incorporation of Ag enhanced electron transfer from Co_3_O_4_ to Ag, further improving OER performance and achieving a low overpotential of 206 mV at a current density of 10 mA cm^−2^. Tian et al. [97] demonstrated that an Ru–Co_3_O_4_ heterojunction containing only 0.55 wt% of Ru exhibited notably enhanced bifunctional activities. This electrocatalyst exhibited remarkably low overpotential values of 11 mV for the HER and 253 mV for the OER at a current density of 10 mA cm^−2^, achieving a cell potential of 1.49 V when applied in an electrolyzer. The bifunctional performance of Co_3_O_4_-based composites with various materials, as reported in the literature, is summarized in Table 3.

From the analysis of previously designed and developed Co_3_O_4_-based composites for bifunctional electrocatalysts, several key points have been identified through a comprehensive evaluation: Carbon derivatives, such as graphene, carbon nanotubes (CNTs), and graphitic carbon nitride (g-C_3_N_4_), significantly enhance the electrical conductivity of Co_3_O_4_-based composites, facilitating efficient electron transfer during electrocatalytic reactions. These materials also offer a high surface area, promoting the uniform dispersion of Co_3_O_4_ nanoparticles and increasing the exposure of active sites, while providing structural support to the composite. Co_3_O_4_-carbon composites can be synthesized using various methods, including hydrothermal/solvothermal processes, chemical vapor deposition (CVD), and electrochemical deposition. However, the fabrication process often involves complex and multistep procedures, such as hydrothermal treatments, pyrolysis, or chemical functionalization, which are time-consuming and require precise control. Additionally, certain carbon derivatives, like g-C_3_N_4_ and activated carbon, require high-temperature pyrolysis for preparation, leading to increased energy consumption and production costs. The large-scale application of high-performance carbon derivatives, such as reduced graphene oxide (rGO) or CNTs, is further limited by their high cost. Developing cost-effective and scalable synthesis methods and exploring alternative carbon sources could help overcome these challenges and optimize their real-world applicability. Another notable drawback of carbon derivatives in composites is their susceptibility to degradation under harsh electrolytic conditions. In such environments, the oxidation of carbon can lead to the formation of CO or CO_2_, reducing the composite’s stability and longevity. Addressing these issues through innovative design and material engineering could unlock the full potential of Co_3_O_4_-carbon composites in electrocatalytic applications.

MOFs are highly beneficial in composite fabrication, offering enhanced surface area and tunable porosity, which enable efficient dispersion of Co_3_O_4_ nanoparticles and improved mass transfer during electrocatalytic reactions. Additionally, MOFs with specific metal ions and organic linkers allow precise control over the composition and morphology of Co_3_O_4_-based composites, optimizing catalytic properties. However, MOF usage involves challenges such as multistep synthesis processes requiring strict control of temperature, pressure, and solvent selection. The reliance on rare or highly pure organic linkers can increase costs, and MOFs’ thermal sensitivity necessitates high-temperature calcination to form stable Co_3_O_4_ structures, potentially compromising structural integrity. Furthermore, the use of organic solvents and linkers raises environmental and safety concerns, particularly for large-scale applications. MOF-derived Co_3_O_4_ composites may also experience structural degradation or reduced activity under harsh operational conditions, limiting their long-term stability and efficiency. Co^3^O^4^-based composites with metal oxides, noble metals, heteroatoms, and sulfides offer a versatile platform to enhance material performance for various applications. However, challenges in scalability exist, and selecting the optimal combination and synthesis method depends on the specific application and desired properties.

## 4. Conclusions

In recent years, Co_3_O_4_-based composites have emerged as promising candidates for electrocatalytic water splitting owing to their favorable electrochemical properties, environmental benignity, and cost-effectiveness. By combining Co_3_O_4_ with materials such as metal oxides, carbon derivatives, and MOFs, researchers have successfully enhanced its catalytic performance, particularly by reducing its overpotential, improving its stability, and accelerating its reaction kinetics. Moreover, fabrication strategies such as nanostructuring and doping have further refined these composites, resulting in notable advancements in HER and OER activities. These developments are promising for increasing the efficiency of sustainable energy conversion technologies. However, despite the progress achieved, further advancements are required to fully harness the potential of Co_3_O_4_-based composites in commercial-scale water-splitting applications.

## 5. Limitations

Despite significant advancements in Co_3_O_4_-based composites, several challenges remain:

1. Stability and Durability: the prolonged operation of Co_3_O_4_-based catalysts in harsh electrochemical environments often leads to degradation, posing a major obstacle to ensuring long-term stability.
2. Optimization of Catalytic Performance: although improvements have been made, the catalytic performance of Co_3_O_4_ composites still falls short compared to other best-performing precious metal-based catalysts.
3. Scalability: developing scalable synthesis methods for Co_3_O_4_-based composites remains a critical challenge, particularly in producing catalysts with consistent and reliable properties for industrial applications.
4. Mechanistic Understanding: while research on Co_3_O_4_ composites is expanding, the exact mechanisms driving their enhanced catalytic activity remain unclear. Further investigations are needed to clarify these processes at the atomic level.

## 6. Future Prospects

The future of Co_3_O_4_-based composites for water splitting holds substantial promise, with several potential directions likely to drive further advancements:

1. Advanced Material Design: the exploration of novel composite structures, such as heterostructures and core–shell architectures, could unlock new levels of catalytic performance.
2. Integration with Other Technologies: integrating Co_3_O_4_-based composites with emerging technologies, such as solar-driven water splitting or hybrid energy systems, could pave the way for integrated renewable energy solutions.
3. In situ Characterization Techniques: the development of advanced characterization methods, particularly in-situ and operando studies, can provide deeper insights into catalytic mechanisms, enabling the rational design of more efficient and durable catalysts.
4. Sustainability and Cost Reduction: efforts to optimize low-cost, scalable synthesis methods will be critical to ensuring the economic viability of Co_3_O_4_-based composites for industrial-scale applications.
5. Computational Modeling and Machine Learning: harnessing computational tools and machine learning techniques could substantially accelerate the discovery and optimization of Co_3_O_4_-based materials with enhanced properties.

By addressing these challenges and exploring new opportunities, Co_3_O_4_-based composites have the potential to make a profound impact on electrocatalytic water splitting and renewable energy technologies.

## Figures and Tables

**Table 1:** Bifunctional (HER–OER) performance of Co_3_O_4_-based composites with different carbon derivatives.

Composite Material	Electrolyte	η (mV)/Current Density (mA cm^−2^)		Tafel Slope (mV dec^−1^)		Reference
		OER	HER	OER	HER	
Co_3_O_4_/CC	0.1 M KOH (OER) 0.1 M H_2_SO_4_ (HER)	357/10	291/10	283	202	[56]
Fe-Co_3_O_4_/CNT	1 M KOH	300/10	120/10	62	54	[61]
Co_3_O_4_/g-C_3_N_4_	1 M KOH	170/20	151/20	188	176	[62]
C–Co_3_O_4_	1 M KOH	250/10	163/10	54	89	[63]
Co_3_O_4_/Ppy/MWCNT	0.1 M KOH	340/10	490/10	87	110	[64]
Co_3_O_4_/WO_3_/C	1 M KOH (OER) 0.5 M H_2_SO_4_ (OER & HER)	229/10 295/10	123/10	63 77	36	[65]
Co_3_O_4_/MoO_3_/g-C_3_N_4_	1 M KOH (OER) 0.5 M H_2_SO_4_ (HER)	206/10	125/10	60	94	[66]
Co_3_O_4_/CF	1 M KOH	155/10	380/10	101	116	[67]
Co_3_O_4_/graphene	1 M KOH	283/10	108/10	25	90	[70]
Co_3_O_4_–carbon microtubes	1 M KOH	158/10	210/10	-	-	[71]
N–Co_3_O_4_@C@NF	1 M KOH	96/10	42/10	89	56	[72]

**Table 2:** Bifunctional (HER–OER) performance of previously reported MOF-derived Co_3_O_4_-based composites.

Composite Material	Electrolyte	η (mV)/Current Density (mA cm^−2^)		Tafel Slope (mV dec^−1^)		Reference
		OER	HER	OER	HER	
Au@C/Co_3_O_4_	1 M KOH	452/10	482/10	74.7	76.1	[22]
RuO_2_–Co_3_O_4_ heterojunction	1 M KOH	305/10	89/10	-	-	[30]
Co_3_O_4_–N doped carbon	1 M KOH	318/10	106/10	97	109	[73]
Ni/Co/Co_3_O_4_@C	1 M KOH	246/30	143/30	50.2	114	[74]
Co_3_O_4_/PPy	1 M KOH	220/10	140/10	57.7	83	[75]
Co_3_O_4_@Co/NCNT	0.5 H2SO4	161/10	171/10	-	121	[76]
Co@Co_3_O_4_/FeNS–rGO	1 M KOH	287/10	130/10	69	111	[77]
Co_3_O_4_/MoS_2_	1 M KOH	230/20	205/10	45	89	[78]
Co_3_O_4_@Mo-Co_3_S_4_–Ni_3_S_2_/NF	1 M KOH	295/10	116/10	98	97	[79]
Co/Co_3_O_4_@C	1 M KOH	192/10	357/10	-	-	[80]
P–CoN/CWO/Co_3_O_4_	1 M KOH	175/10	-	98.9	-	[83]
P–CoN/CMO/Co_3_O_4_	1 M KOH	-	109/10	-	89.1	[83]

**Table 3:** Bifunctional (HER–OER) performance of Co_3_O_4_-based composites with materials such as metal oxides, sulfides, hydroxides, precious metals, heteroatoms, and transition metal dichalcogenides.

Composite Material	Electrolyte	η (mV)/Current Density (mA cm^−2^)		Tafel Slope (mV dec^−1^)		Reference
		OER	HER	OER	HER	
PtRu−Co_3_O_4_	0.5 M H_2_SO_4_	143/10	99/10	67	59	[14]
NiCoP@Co_3_O_4_/NF	1 M KOH	120/10	72/10	62.85	56.26	[17]
P-Co_3_O_4_/NF	1 M KOH	260/20	97/10	60	86	[32]
N/S-VO-Co_3_O_4_	1 M KOH	294/10	181/10	71	79	[33]
RuO_2_@Co_3_O_4_	1 M KOH 0.5 M H_2_SO_4_	152/10 219/10	90/10 33/10	68 73	103 95	[34]
CoP/Co_3_O_4_	1 M KOH	257/20	98/10	61.2	53.6	[35]
DV (dual vacancies)-Co_3_O_4_/Co_3_S_4_	1 M KOH	233/100	26/10	75	58	[40]
Ni–Co_3_O_4_ film	1 M KOH	353/10	190/10	51	148	[60]
Electric-field-treated Ni–Co_3_O_4_ film	1 M KOH	311/10	93/10	43	69	[60]
P–Co_3_O_4_	1 M KOH	280/10	120/10	51.6	52	[85]
NiCoO_2_@Co_3_O_4_	1 M KOH	79.9/10	88.2/10	46.2	72.2	[86]
Ni-Co_3_O_4_ NFs	1 M KOH	330/10	74/10	97	52	[87]
WCoSe/WCo_3_O_4_	1 M KOH	175/10	98/10	62	72	[88]
W-Co_3_S_4_@Co_3_O_4_	1 M KOH	260/10	140/10	39.8	94	[89]
Co_3_O_4_–MoSe_2_@C	1 M KOH	360/10	144/10	75.7	95.5	[90]
Co_3_O_4_@NiO	1 M KOH	156/10	65/10	101	61	[91]
Mo–Co_3_O_4_/NC	1 M KOH	276/10	91/10	66.7	139.7	[92]
FeOOH@Co_3_O_4_	1 M KOH	370/10	174/10	71	57	[93]
P–Co_3_O_4_	1 M KOH	310/20	111/20	122.5	136.5	[94]
Ru-Co_3_O_4_	1 M KOH	270/100	48.6/100	103.8	27.7	[95]
Co_3_O_4_–Ag	1 M KOH	206/10	51/10	55	49	[96]
Ru/Co_3_O_4_ heterojunction	1 M KOH	253/10	11/10	77	51	[97]
RuO_2_–Co_3_O_4_	1 M KOH	260/10	75/10	73	54.4	[98]

## Data Availability

No new data were created.
